# High efficacy and safety of CD38 and BCMA bispecific CAR-T in relapsed or refractory multiple myeloma

**DOI:** 10.1186/s13046-021-02214-z

**Published:** 2022-01-03

**Authors:** Yuanyan Tang, Haisen Yin, Xinying Zhao, Dan Jin, Yan Liang, Tao Xiong, Lu Li, Wen Tang, Jiangzhao Zhang, Min Liu, Zhuojun Yu, Huimin Liu, Sibin Zang, Zhiping Huang

**Affiliations:** 1 Department of Hematology, Jingzhou Central Hospital, The Second Clinical Medical College, Yangtze University, No.60, Jingzhong Road, Jingzhou, 434020 Hubei Province China; 2 Department of Gastroenterology, Key Laboratory of Hubei Province for Digestive System Diseases, Renmin Hospital of Wuhan University, Wuhan, China; 3 Cellyan Therapeutics Co. Ltd, Wuhan, China

**Keywords:** Multiple myeloma, BCMA, CD38, CAR-T, Efficacy, Safety

## Abstract

**Background:**

B-cell maturation antigen (BCMA) chimeric antigen receptor T (CAR-T) cell therapy has obtained promising results in relapsed or refractory multiple myeloma (R/R MM), while some patients do not response, or relapse in short term after treatment. Combining with anti-CD38 might solve the problem of targeting BCMA alone. We aimed to assess the efficacy and safety of BCMA and CD38 (BCMA-CD38) bispecific CAR-T cells in R/R MM patients.

**Methods:**

We did a single-center, single-arm clinical study at the Second Affiliated Hospital of Yangtze University in China. Patients meeting with the inclusion criteria were administered with fludarabine and cyclophosphamide before CAR-T cells infusion. Response and adverse events were assessed after infusion. This study was registered with the Chinese Clinical Trial Registration Center (ChiCTR1900026286).

**Results:**

First, we found BCMA-CD38 CAR-T cells exhibited enhanced killing effect on BCMA+CD38+ cells in vitro, compared to BCMA CAR-T and CD38 CAR-T cells*.* We further demonstrated its anti-tumor activity in vivo. Then, we enrolled 16 R/R MM patients for safety and efficacy analyses. Of the 16 evaluable patients, 14 (87.5%) respond to the treatment, including 13 stringent complete response (sCR) and one partial response (PR), while two patients did not respond. At a median follow-up of 11.5 months, of the 13 patients who achieved sCR, 76.9% (10/13) did not relapse or progress during follow-up. Relapse occurred in 3 patients (Patient 2, 3 and 4) after achieving sCR. In sum, four patients died, of which one died of hemophagocytic lymphohistiocytosis syndrome secondary to severe cytokine release syndrome (CRS) and three died of disease progression or relapse. The 1-year progression-free survival rates was 68.8%. The 1-year overall survival rate was 75.0%. Extramedullary lesions were eliminated in 62.5% (5/8) patients. The most common symptoms after CAR-T infusion were cytopenia (16, 100%), fever (10, 62.5%), fatigue (8, 50.0%) and myalgias (8, 50.0%). Twelve patients (75.0%) were observed with various grades of CRS, of which five patients (31.3%) got serious CRS (Grade ≥ 3). The CAR+ cell expansion levels were associated with the severity of CRS. Transient clonal isotype switch was observed after CAR-T infusion.

**Conclusion:**

Our results confirm that BCMA-CD38 CAR-T cells therapy is feasible in treating R/R MM patients, with high response rate, low recurrence rate and manageable CRS, which will be a promising treatment option for R/R MM.

**Trial registration:**

ChiCTR1900026286, registered on September 29, 2019, retrospectively registered, URL: https://www.chictr.org.cn/showproj.aspx?proj=43805

**Supplementary Information:**

The online version contains supplementary material available at 10.1186/s13046-021-02214-z.

## Background

Multiple Myeloma (MM) is an almost incurable plasma cell malignancy, and most patients will eventually develop refractory disease and suffer a fatal relapse [1], in spite of the application of new drugs [2–4]. Recent studies have verified the activity of chimeric antigen receptor T (CAR-T) cell therapies in the treatment of relapsed or refractory MM (R/R MM), but the results established vastly different [5–7].

B-cell maturation antigen (BCMA), also known as CD269, is a member of the tumor necrosis factor superfamily, which is mainly expressed on plasma cells, and plasmacytoid dendritic cells, but not other normal cells, considered to be an ideal target for CAR-T cells in the treatment of R/R MM [8–10]. Many clinical trials of anti-BCMA CAR-T cell in R/R MM have been registered. Idecabtagene Vicleucel (Idel-cel) has been approved for treating R/R MM, based on a phase II study that 73.4% of patients had a response and 31.3% had a complete response or better [11]. Ciltacabtagene autoleucel (Cilta-cel) is being reviewed with overall response rate (ORR) of 94.8%, stringent complete response (sCR) of 55.7%, and very good partial response rate (VGPR) of 32.0% [12]. However, the efficacy of targeting BCMA have showed promising but varied ORR ranging from 30 to 100%, or relapse occurs in a short time after CAR-T therapy, due to clonal evolution and loss of subclone in tumor cells, immunosuppression in bone marrow microenvironment, or exhaustion of CAR-T cells etc. [13, 14]. Besides anti-BCMA CAR-T cell therapy, anti-CD138, targeting κ light chains, or anti-NKG2D CAR-T therapy did not achieve an ideal efficacy in causing remission of R/R MM [15] [16] [17]. Despite many advantages compared to other CAR-T immunotherapy against R/R MM, anti-BCMA CAR-T therapy still need to further improve progression-free survival (PFS) and reduce side effects. It is expected that expanding the coverage of MM cells will achieve more favorable duration responses and less relapse by preventing single target ineffectiveness or overcoming BCMA negative relapse [18].

Daratumumab, as the first monoclonal antibody directly targeting MM cells, has been approved by the United States Food and Drug Administration for treating R/R MM. It has broad-spectrum killing activity, targeting the transmembrane extracellular enzyme CD38 molecule that highly expressed on the surface of MM cells, which can induce rapid apoptosis of tumor cells through a variety of mechanisms, prolong the survival of patients, and has no serious inhibition of myeloid cell growth [19]. Moreover, CD38 antigen has been optimized as a target of therapeutic CAR-T cells for MM in a preclinical study [20].

In this study, we first confirmed the enhanced killing effect of BCMA-CD38 CAR-T cells compared to BCMA CAR-T in vitro experiments, and further demonstrated its anti-tumor activity in vivo. On basis of these preliminary data, we recruited 16 R/R MM patients, aiming to detect the efficacy and safety of BCMA-CD38 CAR-T cells in R/R MM patients.

## Methods

### CAR-T production

The CAR fusion gene was inserted into third generation lentiviral cytoskeleton vector pLVX-EF1. PLVX-EF1 with CAR fusion gene, the helper plasmid and lentivirus packaging were co-transfected into HEK-293 T cells. The virus was then harvested and purified [21]. Peripheral blood mononuclear cells (PBMC) were isolated from 60 to 100 ml of peripheral blood collected from subjects by density gradient centrifugation (Ficoll Paque, GE life sciences). The initial cells were stimulated with CD3 and CD28 antibodies (0.5 μg/ml, R&D) and IL-2 (200 IU/ml, T&L) on day 0, then transduced with the CAR encoded lentivirus (MOI = 5:1) on day 2 to obtain CAR-T cells. After cultured for another 7 to 12 days, the cells were assessed by flow cytometry (FCM) of stained for BCMA-scFv with recombinant BCMA protein (rBCMA protein, ACRO) for transduction efficiency. The cells were also qualified evaluated for sterility, endotoxin and cell activity before infusion.

### CAR-T function studies in vitro

To demonstrate the killing effect of CAR-T cells, we used an adherent cell line (BCMA+CD38+ Hela) for *vitro* experiments. BCMA+CD38+ Hela cells (1 × 10^4^ per well) were seeded into the special culture in real-time unlabeled cell analysis (RTCA) system, with conductive electrode at the bottom of the culture plate. Three replicates were done for each sample. Eighteen hours after inoculation, tumor cells were quantitatively treated by T cells or CAR-T cells at different effective to target ratios (E:T) for another 42 h. The conductivity indices of the culture plates were collected every 30 min to record the relative numbers of tumor cells.

### In vivo xenograft experiments

Four-week-old NOD-SCID mice (NOD-Prkdc^scid^-Il2rg^null^) were purchased from the National Resource Center for Rodent Laboratory Animals of China. BCMA+CD38+ K562 luciferase cells (2 × 10^6^ each), which were suspended in 100 μl of PBS, were administered to NOD-SCID mice via tail vein injection. On day 7, mice bearing engrafted tumors were randomized to treat with 2 × 10^7^ mock-transduced T cells or BCMA-CD38 transduced T cells via tail-vein injection (*n* = 6 each group). Tumor burden was monitored by bioluminescence imaging using an IVIS Lumina III LT Imaging System (PerkinElmer) on day 7, 14, and 28. The ending point was either death of the mice or 36 days after injection. Peripheral blood and bone marrow samples were collected from all surviving mice for FCM tests. After lysis of red blood cells, GFP and anti-Human CD45 were used to label the tumor cells. All experiments were carried out as approved by the Institutional Animal Care and Use Committee of the Second Affiliated Hospital of Yangtze University.

### Clinical trial design and participants

We did a single-center, single-arm, investigator initiated clinical study at the Second Affiliated Hospital of Yangtze University in China. This study was approved by the institutional independent ethics committee (reference number: 2017–010-02) of the Second Affiliated Hospital of Yangtze University in accordance with the Declaration of Helsinki, and was registered with Chinese Clinical Trial Registration Center (ChiCTR1900026286). All patients provided written informed consent. The primary objective is to evaluate the safety of BCMA-CD38 CAR-T cells, and the secondary objective is to evaluate the anti-myeloma response of the treatment.

Patients were eligible if they were 18–80 years of age, histologically confirmed MM, BCMA and CD38 positive confirmed by FCM, and met the International Myeloma Working Group (IMWG) diagnostic criteria for MM [22]. R/R MM patients should meet: treatment failure or disease progressed after 2 or more prior lines of treatment regimens, and disease progression or relapse is defined as IMWG. Adequate renal, hepatic, lung and heart function were required, and detailed definitions were described in Additional file 1. Previous BCMA or CD38 targeted cell therapy was not allowed. Detailed inclusion and exclusion criteria are provided in Additional file 1.

### Procedures

All patients were confirmed as having MM after analysis of histology, immunology, imageological diagnosis, SPEP and UPEP, and had been categorized into different stages according to international stage system (ISS).

All patients underwent peripheral blood collection to obtain PBMC from which T cells were purified. After 9–14 days of CAR-T cells preparation, BCMA-CD38 CAR-T cells were infused. Three doses of fludarabine (25 mg/m^2^) and cyclophosphamide (250 mg/m^2^) were administered daily on days − 4, − 3 and − 2 before CAR-T cells infusion. After the infusion of the engineered BCMA-CD38 CAR-T cells, all the patients were followed up at regular intervals.

### Assessment

The objective is to evaluate the safety and efficacy of BCMA-CD38 CAR-T therapy in R/R MM. The evaluation of response was according to the IMWG criteria for response and minimal residual disease assessment in multiple myeloma (2016) [23], including sCR, complete response (CR), VGPR, partial response (PR), minimal response (MR), stable disease (SD), progression disease (PD). The response evaluation included the number of plasma cells in the bone marrow determined by morphology and minimal residual disease (MRD), FCM, serum protein electrophoresis (SPEP), urine protein electrophoresis (UPEP), and serum free immunoglobulin light chains. Magnetic resonance imaging (MRI) or computed tomographic (CT) was employed to assess extramedullary involvement. Peripheral blood cells were collected at different time points to measure the percentages of CAR-T cells using FCM.

The cytokine release syndrome (CRS) was graded from 0 to 5 according to the Penn grading [24]. The severe CRS was defined as grade 3 or higher. In addition, interleukin-6 (IL-6), c-reactive protein (CRP) and ferritin were employed to help evaluate the severity of CRS.

### Statistical analysis

We used student’s *t-test* to compared variable in vitro and in vivo experiments. The Mann-Whitney test was used to compare continuous variables.

## Results

### BCMA-CD38 bispecific CAR-T effectively lysed BCMA+CD38+ tumor cells in vitro and in vivo studies

**Fig. 1:**
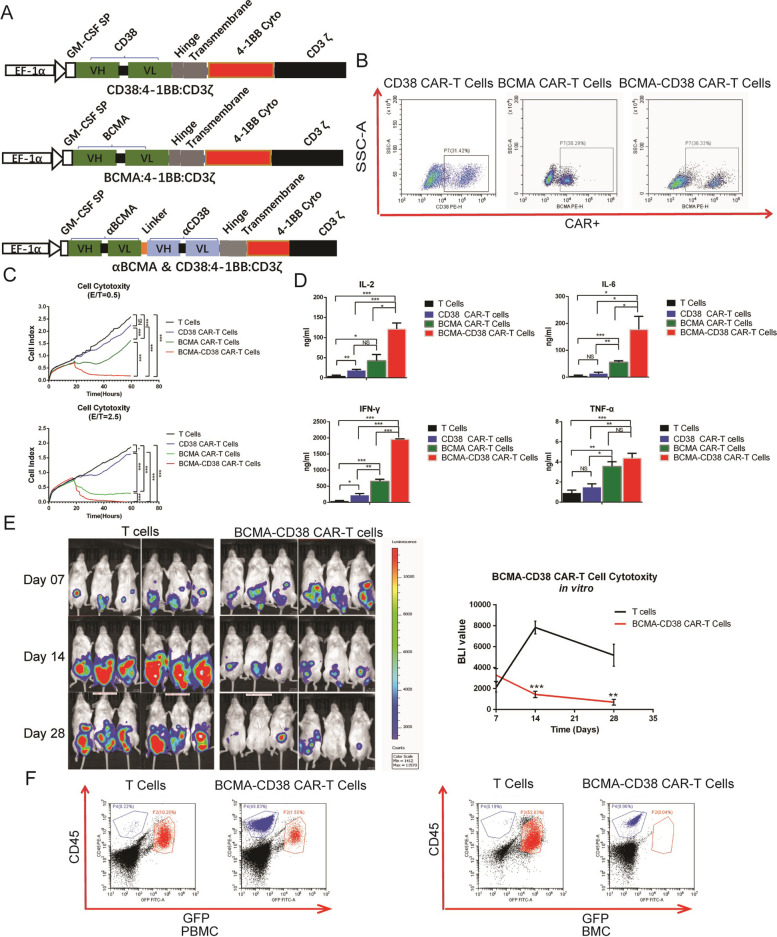
BCMA-CD38 bispecific CAR-T effectively lysed BCMA+CD38+ tumor cells in vitro and in vivo studies. **A** Diagram of CAR cDNA plasmid and CAR-T cell structures. BCMA-CD38 CAR was composed of BCMA and CD38 targeting domains, human CD8 alpha hinge and transmembrane domain (CD8α hinge + TM), human 4-1BB cytoplasmic domain, and a human CD3 zeta cytoplasmic domain (CD3ζ). BCMA or CD38 CAR was composed of BCMA or CD38 targeting domains, human CD8 alpha hinge and transmembrane domain (CD8α hinge + TM), human 4-1BB cytoplasmic domain, and a human CD3 zeta cytoplasmic domain (CD3ζ). **B** Representative dot-plots of FCM analysis, detecting the CAR+ expression in T cells 6 days after CD38, BCMA and BCMA-CD38 virus transduction. **C** Cell indices of BCMA+CD38+ Hela cells were recorded by RTCA after treated with T cells, CD38 CAR-T cells, BCMA CAR-T cells and BCMA-CD38 CAR-T cells at E:T ratios of 0.5 and 2.5. Three replicates were done for each sample. Area under curves (AUC) of cell indices were calculated and statistically analyzed. NS: no statistically significant, *P < 0.05, **P < 0.01, ***P < 0.001. **D** IL-2, IL-6, IFN-γ and TNF-α levels were compared after treated with T cells, CD38 CAR-T cells, BCMA CAR-T cells and BCMA-CD38 CAR-T cells at E:T ratios of 2.5. NS: no statistically significant, *P < 0.05, **P < 0.01, ***P < 0.001. **E** Bioluminescence imaging (BLI) was used to assess tumor progression and regression of mice at different time after being treated with T cells and BCMA-CD38 CAR-T cells. Quantification of BLI as curves was presented. **P < 0.01, ***P < 0.001. **F** Representative dot-plots of FCM analysis, detecting the percentage of tumor cells in mice treated with T cells and BCMA-CD38 CAR transducing T cells on day 36. The cells were stained with GFP and anti-Human CD45. The blue populations were CAR-T cells expressing human CD45, and the red populations were MM cells expressing GFP and human CD45

CAR was comprised of three main domains: a tumor associated antigen binding region, CD8α hinge and transmembrane region, and 4-1BB and CD3z intracellular motif (Fig. 1A). The CD38 antibody with 1000-fold reduced affinity was screened from plenty of CD38 antibodies with a broad range of different affinities. The CAR gene was exogenously expressed on the surface of CD3+ T cells. FCM analysis demonstrated that the proportion of CAR+ T cells on the 6th day of CD38, BCMA and BCMA-CD38 CAR virus infection could reach 31.42, 38.29 and 36.33%, respectively (Fig. 1B). The lysis effect of T cells, CD38 CAR-T cells, BCMA CAR-T cells and BCMA-CD38 CAR-T cells in vitro was recorded by conductive electrode at an E:T ratio of 0.5 and 2.5. BCMA+CD38+ Hela cells treated with BCMA-CD38 CAR-T cells were more severely suppressed, compared to BCMA CAR-T and CD38 CAR-T (Fig. 1C). CD38 CAR-T cells showed very weak killing effect for low affinity. Generally, more cytokines (IL-2, IL-6, IFN-γ and TNF-α) were detected in cells treated by BCMA-CD38 CAR-T cells, compared to BCMA CAR-T cells (Fig. 1D). These results demonstrated that BCMA-CD38 CAR-T cells exhibited stronger lyses effect than BCMA CAR-T cells and CD38 CAR-T cells. As for in vivo experiments, mice received 3 × 10^6^ T cells (*n* = 6) and BCMA-CD38 CAR-T cells (*n* = 6) 7 days after tumor cells injection, respectively. Bioluminescence imaging (BLI) showed a lower tumor burden in mice treated with BCMA-CD38 CAR-T cells as compared with T cells on d14 (*P* < 0.001) and d28 (*P* = 0.002) (Fig. 1E). During the experiment, we observed mild symptoms including lethargy, decreased activities and wrinkle in skin in two groups. And the symptoms of the two groups did not show much different, suggesting that there was no obvious CD38 off-target toxicity in xenograft models. By FCM analyses, tumor cells stained with GFP and anti-human CD45 of both PBMC (10.28% vs. 1.50%) and bone marrow cells (BMC) (52.63% vs. 0.04%) were significantly reduced in mice treated with BCMA-CD38 CAR-T cells, compared with those treated with T cells on d36. In contrast, T cells stained with anti-Human CD45 on d36 rose to 49.83% in PBMC and 9.96% in BMC, respectively. To sum up, BCMA-CD38 bispecific CAR-T showed strong anti-tumor activity in vitro and in vivo. Hence, we then focus on investigating the safety and toxicity of BCMA-CD38 CAR-T in R/R MM patients.

### Patient characteristics

To further demonstrate its efficacy in clinical, 16 R/R MM patients were enrolled (Supplementary Fig. 1). The study was initiated on May 20, 2018, and the cutoff date is Jun 1, 2020. The characteristics of the patients were list in Table 1. The patients had a median age of 58.5 (range 48–78) years old, and a median of 28.5 months (range 4–72) of disease duration before enrollment. The ISS stage of 81.3% (13/16) patients is stage III. Eight patients (50.0%) had extramedullary infiltration. Eleven patients (68.8%) have cytogenetic abnormalities including 1q21 (10/16, 62.5%), del17p (3/16, 18.8%), RB1 (1/16, 6.3%), and D13S319 (1/16, 6.3%). The median clonal sizes of Del17p and 1q21 were 20 and 25%, respectively. Clonal sizes of the cytogenetic abnormalities in detail were shown in Table 1. Three (18.8%) patients had received autologous hematopoietic stem cell transplantation (ASCT) before enrollment. The median percentages of MRD in BMC were 5.75%.

**Table 1:** Clinical Characteristics of patients with BCMA-CD38 CAR-T at baseline

NO.	Age	Months of MM	Stage (ISS)	Subtype	EM	Cytogenetic abnormalities	Prior treatments				MRD (%)
							Prior lines	PI	IMID	ASCT	
1	62	6	III	κ	No	1q21 (9.0%)	2	BTZ		No	6.96
2	60	60	III	IgA-λ	No	1q21 (14.0%), RB1 (13.0%), D13S319 (13.0%)	3	BTZ	Thal, Lenal, Pomal	Yes	3.68
3	50	18	I	IgG-κ	No	1q21 (31%), Del17p (13%)	2	BTZ		Yes	8.80
4	78	72	III	IgA-λ	Yes	1q21 (26.0%), Del17p (20.0%)	3	BTZ	Thal, Lenal	No	13.50
5	70	66	I	κ	No	Del17p (24.0%)	3	BTZ	Lenal Thal	No	9.34
6	66	14	III	IgG-λ	Yes	1q21 (12%)	3	BTZ	Lenal	No	32.21
7	57	46	III	λ	No	No	3	BTZ,	Thal	No	4.26
8	57	33	III	λ	Yes	No	3	BTZ	Thal	No	3.22
9	64	43	III	IgG-κ	No	No	3	BTZ	Thal, Lenal	No	0.95
10	55	10	III	IgA-κ	No	No	2	BTZ		No	4.31
11	74	4	III	IgA-κ	Yes	No	2	BTZ		No	33.00
12	63	42	III	IgG-κ	Yes	1q21 (62.0%)	2	BTZ		No	5.46
13	57	5	III	IgA-λ	Yes	1q21 (24.0%)	2	BTZ	Thal	No	21.04
14	53	39	II	IgG-λ	Yes	1q21 (27.0%)	3	BTZ	Thal, Lenal Pomal	No	2.92
15	48	24	III	IgG-κ	Yes	1q21 (11.2%)	3	BTZ		Yes	5.03
16	55	7	IIII	IgA-κ	No	1q21 (45.0%)	3	BTZ		No	6.04

*ISS* International stage system, EM Extramedullary lesions, *PI* Proteasome inhibitor, *IMID* immunomodulatory agents, *ASCT* Autologous hematopoietic stem cell transplantation, *BTZ* Bortezomib, *THAL* Thalidomide, *Lenal* Lenalidomide, *Pomal* Pomalidomide, *MRD* Minimal residual disease

### BCMA-CD38 CAR-T was effective in alleviating intra- and extramedullary infiltration

After a median of 12 days (9–14) of preparation, the total numbers of cultured cells reached a median number of 7.1 × 10^8^ (1.0–35.7 × 10^8^) (Table 2). The percentage of infusion T cells that expressed CAR-BCMA-CD38 was determined by FCM before infusion, ranging from 9.0 to 50.0% (Table 2). Median dose of CAR+ T cells were 2.1 × 10^6^/kg (0.5–10.0 × 10^6^/kg) (Table 2). A total dose of CAR-T cells was split into two or 3 days for infusion.

**Table 2:** Information of CAR-T cells in production

NO.	Preparation days	T cells (×10^8^)	Fraction of CAR+ T cells (%)	CAR+ T cells ×10^6^/kg
1	10	2.4	18.0	1.0
2	12	35.7	15.0	10.0
3	13	6.7	9.0	1.0
4	12	5.6	14.7	1.6
5	10	3.2	18.0	1.0
6	10	15.7	10.0	2.7
7	13	7.3	30.0	4.0
8	13	24.7	10.0	4.1
9	12	1.0	30.0	0.5
10	12	10.7	15.0	2.1
11	14	6.8	15.0	2.1
12	14	9.5	15.0	2.0
13	13	4.2	18.0	1.0
14	11	3.6	50.0	3.0
15	9	12.0	20.0	4.0
16	10	16.5	10.0	2.9
Median	12	7.1	15.0	2.1

**Fig. 2:**
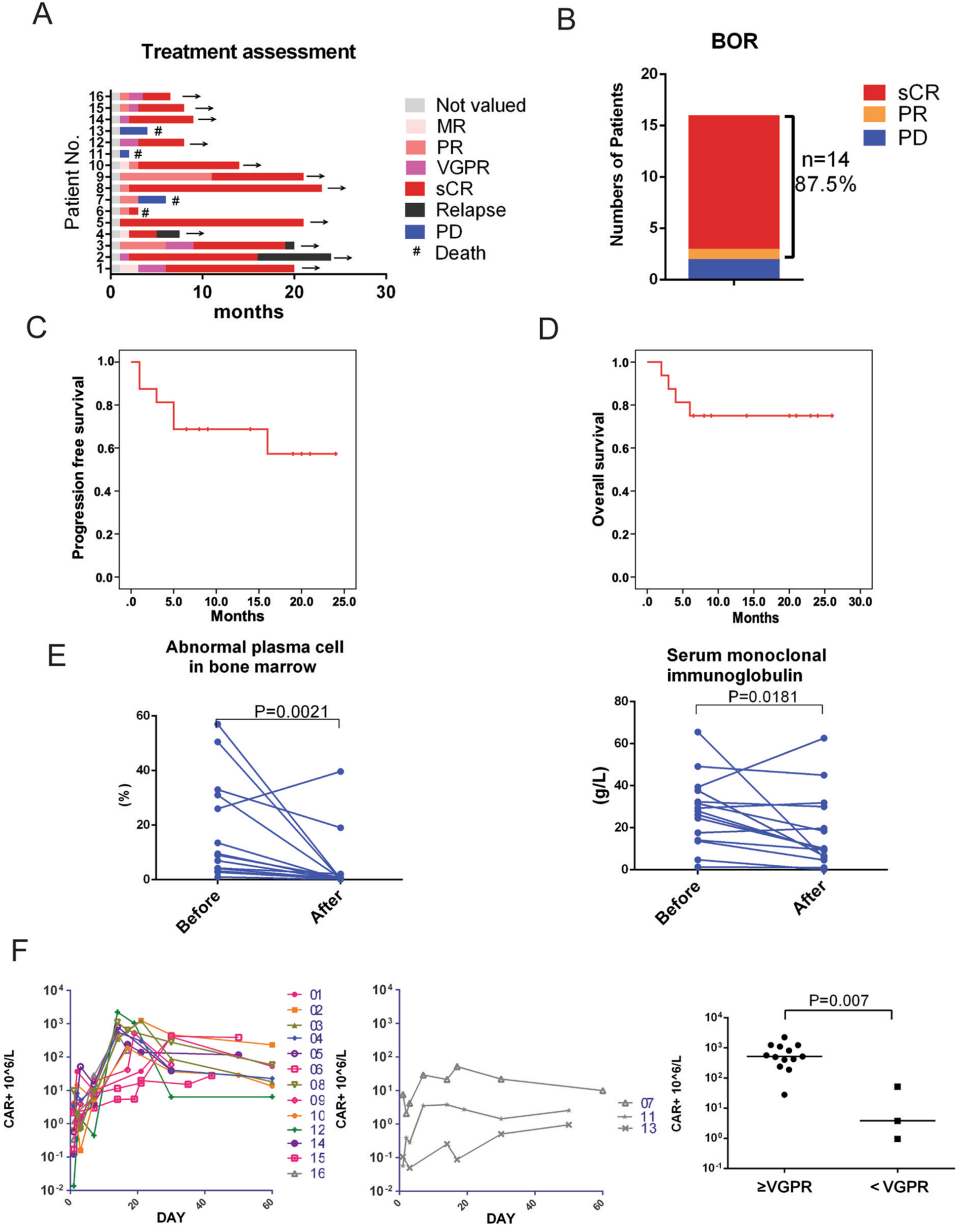
BCMA-CD38 CAR-T cells had potent activity against intramedullary lesion in MM patients. **A** The duration of response to BCMA-CD38 CAR-T in 16 cases was assessed according to IMWG 2016. **B** The best objective response (BOR) after BCMA-CD38 CAR-T infusion to the cut off data. **C** The PFS curves of patients after BCMA-CD38 CAR-T infusion. **D** The OS curves of patients after BCMA-CD38 CAR-T infusion. **E** Left: CD38 expression by FCM in bone marrow before and 1 month after CAR-T cell infusion was compared. Right: Serum MIg determined by SPEP before and 1 month after CAR-T cell infusion was compared. Wilcoxon matched-pairs signed rank test was used for statistical comparison. **F** Left: The amplification curves of CAR+ T cells in peripheral blood of patients (response ≥VGPR) assessed by FCM; Middle: The amplification curves of CAR+ T cells in peripheral blood of patients (response <VGPR) assessed by FCM; Right: Peak values of CAR+ T cells in peripheral blood of patients assessed by FCM were compared between patients whose response ≥VGPR and response <VGPR after CAR-T delivery

According to IMWG 2016 criteria, we evaluate the response of patients (Fig. 2A), and obtained the best objective response (BOR) of every patient (Fig. 2B). Of the 16 evaluable patients, 14 (87.5%) had an overall response to the treatment, including 13 patients achieving a sCR and one patient achieving a PR, while two patients did not respond (Fig. 2B).

At data cut off, median follow-up was 11.5 months, ranging from 6.0 to 26.0 months. Of the 13 patients who achieved sCR, 76.9% (10/13) did not relapse or progress during follow-up. Relapse occurred in 3 patients (Patient 2, 3 and 4) after achieving sCR, and these events took place from 5 to 19 months after response (Fig. 2A). CAR-T cells exhaustion occurred in all the three recurrent patients. Patient 3 and 4 relapsed with BCMA and CD38 positive in bone marrow by FCM detection after 19 and 5 months, respectively (Fig. 2A). However, FCM detection suggested BCMA- and CD38+ in bone marrow of patient 2 after 16 months (Fig. 2A). In sum, four patients died, of which one (Patient 6) died of hemophagocytic lymphohistiocytosis syndrome (HLH) secondary to severe CRS and three died of disease progression or relapse (Fig. 2B). The Kaplan–Meier curve showed PFS rates of 68.8% at 12 months (Fig. 2C). The 1-year overall survival (OS) rate was 75.0% (Fig. 2D).

Eradication of plasma cells in bone marrow by CD38 staining in responders generally occurred in 1 month after CAR-T infusion (*P* = 0.002). Reduced level of monoclonal immunoglobulin (MIg) could be observed in 1 month after infusion (*P* = 0.018), while only one of them turned completely negative (Fig. 2E). It generally took a median of 2 (range from 1 to 10) months to get a negative MIg, longer than to eliminate myeloma cells in bone marrow (Fig. 2E). Our results thus indicated that the dual targeted CAR-T was effective in treating bone marrow infiltration of MM cells. The amplification after infusion at different times of CAR+ T cells in peripheral blood of response ≥VGPR and response <VGPR patients were assessed by FCM. CAR-T cells levels in PBMC of patients usually reached a peak at 7 to 30 days (median 15d) after infusion, and CAR-T cells could be detected even 60 days after infusion (Fig. 2F). The peak CAR-T cell levels of patients who obtained anti-myeloma responses of sCR or VGPR (responders) were higher than the peak CAR-T cell levels of patients who obtained outcomes of SD or PD (*P* = 0.007), indicating higher blood CAR+ cell levels were associated with anti-myeloma response (Fig. 2F).

**Fig. 3:**
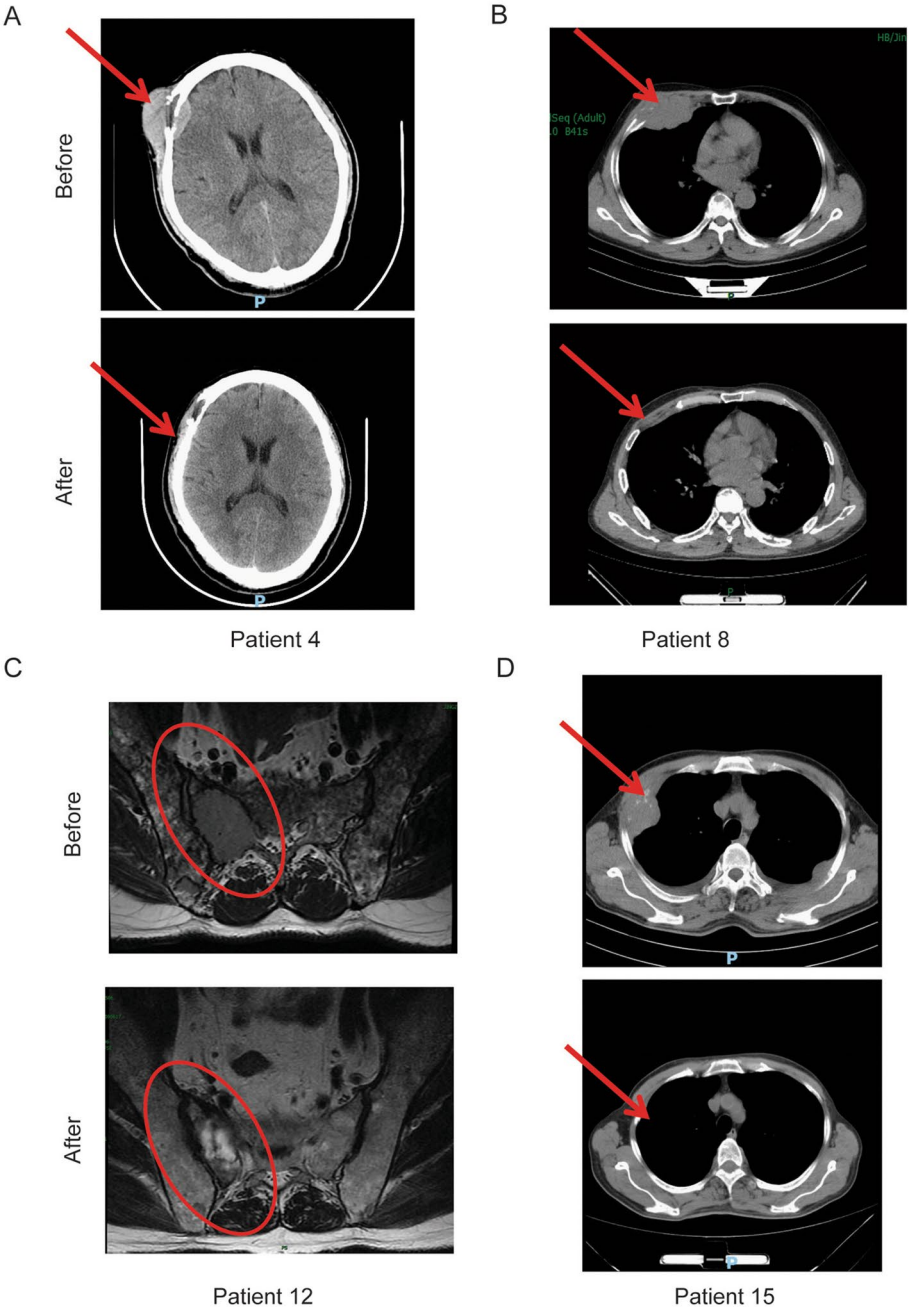
BCMA-CD38 CAR-T cells had potent activity against extramedullary lesions in MM patients. **A** - **D** The extramedullary lesions of patient 4 (**A**), 8 (**B**), 12 (**C**) and 15 (**D**) before and 2 months after BCMA-CD38 CAR-T infusion

Before CAR-T cell infusion, all the patients underwent imaging evaluation. Extramedullary disease was observed in eight patients, and tumor mass in five patients (5/8, 62.5%) was eliminated (Fig. 3A - D, Fig. 6D). As seen on CT imaging, the tumor lesion on the right forehead of patient 4 (Fig. 3A), behind the trachea and within the mediastinum of patient 6 (Fig. 6D), on the thoracic wall of patient 8 and 15 (Fig. 3B and D), next to the sacrum of patient 12 (Fig. 3C) eliminated after CAR-T infusion. Besides, the bone destructions adjacent to these lesions were better repaired than before. Note that masses of the five patients above gradually disappeared were posterior to achieving undetectable MRD. Despite that tumor burden in bone marrow was relieved rapidly 1 month after CAR-T therapy, extramedullary lesions in ethmoid sinus of patient 14 did not eradicate (Supplementary Fig. 2A). Extramedullary infiltration of patient 11 and 13 did not eliminated due to poorly respond to CAR-T therapy (Supplementary Fig. 2B and C). These results demonstrated that BCMA-CD38 CAR-T showed strong activity in eliminating intra- and extramedullary involvement, which also resulted in adjacent bone destruction repaired.

### CRS and its influence factors

**Fig. 4:**
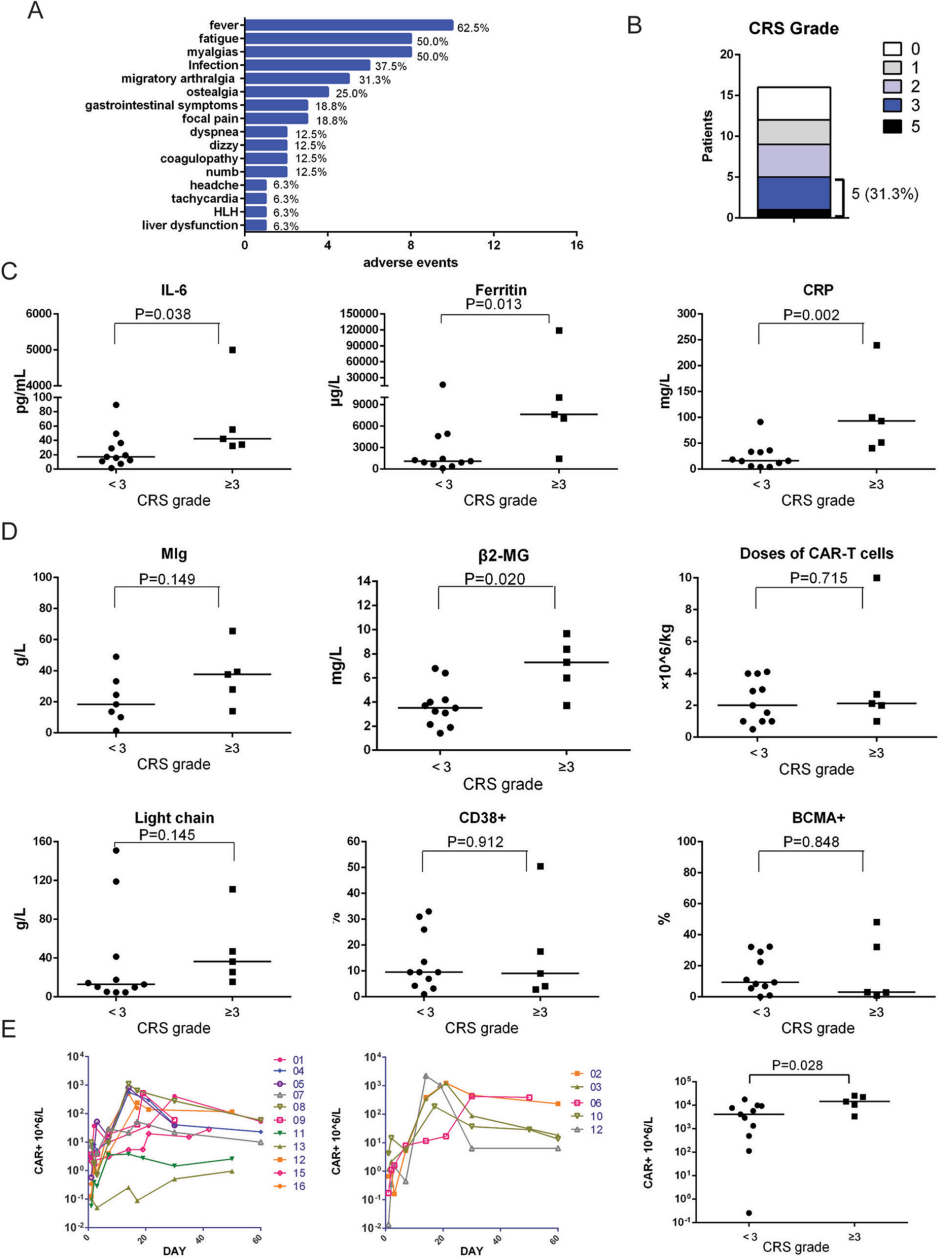
CRS and its risk factors. **A** The adverse events after BCMA-CD38 CAR-T infusion. **B** The rates of different CRS grades. **C** The peak cytokine levels (IL-6, Ferritin and CRP) between CRS grade ≥ 3 and CRS grade <3 were compared. Mann-Whitney test was used for statistical comparison. **D** The risk factors (MIg, β2MG, doses of CAR-T infusion, light chain, and the percentages of CD38+ and BMCA+ plasma in bone marrow) at baseline were compared between CRS grade ≥ 3 and CRS grade <3. Mann-Whitney test was used for statistical comparison. **E** The amplification curves and peak values of CAR+ T cells in peripheral blood of patients assessed by FCM were compared between CRS grade ≥ 3 and CRS grade <3 after CAR-T cells infusion. Mann-Whitney test was used for statistical comparison

We analyzed our cohort to search that 12 patients (75.0%) were observed with various grades of CRS, of which 5 patients (31.3%) had a serious CRS of Grade 3 or more, and one patient (Patient 6) died of severe CRS (Supplementary Table 1, Fig. 4B). Starting day of CRS symptoms typically varied from d0 to d15 (median d3), and can persist 2 to 45 days (median 11.5 d) (Supplementary Table 1). The most common symptoms were fever (10, 62.5%), fatigue (8, 50.0%) and myalgias (8, 50.0%), followed by migratory arthralgia (5, 31.3%), ostealgia (4, 25.0%), gastrointestinal symptoms (3, 18.8%) and focal pain (3, 18.8%) (Fig. 4A). Six patients got infection within 2 months after infusion, of which 3 got lung infection, 1 got digestive infection, 1 got upper respiratory tract infection and 1 got septicemia. We have described the above results in adverse events (Fig. 4A and Supplementary Table 1). Dyspnea, dizzy, numb, liver dysfunction and coagulopathy were also found (Fig. 4A). A high incidence of hematological toxicity was observed in our study (Supplementary Table 2), with 15 (93.8%) patients developing grade 3–5 leukopenia, 6 (37.5%) developing grade 3–4 anemia, and 4 (25.0%) developing grade 3–4 thrombocytopenia. Most of the toxicity was manageable, with three patients being treated with steroids, 11 patients being treated with nonsteroidal anti-inflammatory drugs (NSAIDs), and nine patients being treated with anti-infection. The detailed treatment of CRS was displayed in Supplementary Table 1. However, patient 6 suffered HLH secondary to severe CRS, which is described in detail in Fig. 6. We also measured peak cytokine levels to help evaluated CRS, identifying higher value of IL-6 (*P* = 0.038), Ferritin (*P* = 0.013) and CRP (*P* = 0.002) were observed in CRS grade ≥ 3 (Fig. 4C). We established that β2 microglobulin (β2-MG) as a marker of tumor burden was higher in severe CRS group (*P* = 0.020). However, there is no significant difference of CD38+ or BCMA+ cells in bone marrow, MIg, and light chain between mild and severe CRS (Fig. 4D). These results suggested that β2-MG was an independent predictor of severe CRS. The amplification after infusion at different times of CAR+ T cells in peripheral blood of CRS ≥3 and CRS <3 patients were assessed by FCM. The peak CAR-T cell levels of patients who had severe CRS (CRS ≥3) were higher than the peak CAR-T cell levels of patients who had mild CRS (*P* = 0.028), indicating the CAR+ cell levels were associated with the severity of CRS (Fig. 4E).

### A clonal isotype switch (CIS) presented after CAR-T infusion

**Fig. 5:**
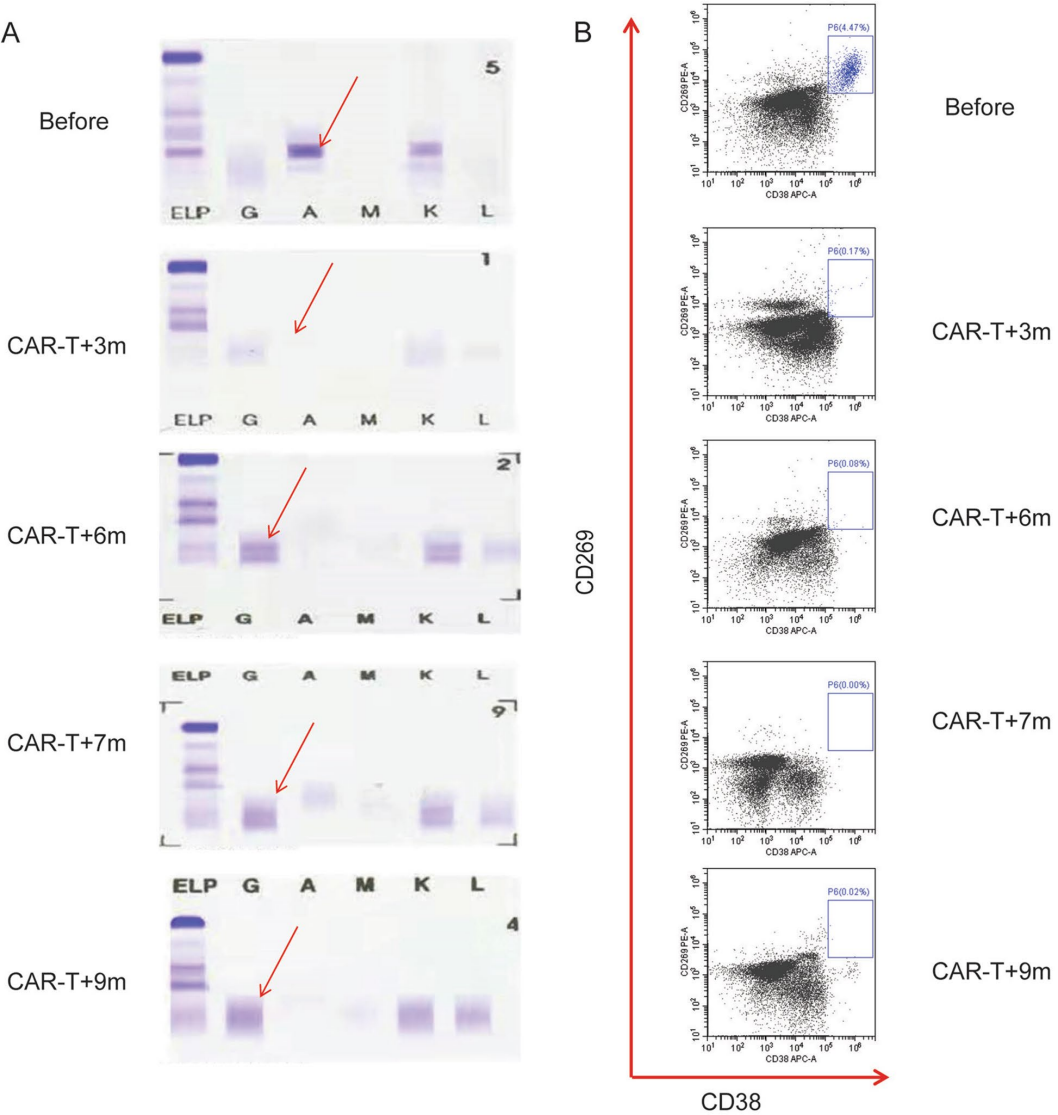
One typical cases of patients treated with BCMA-CD38 CAR-T cells. SPEP (**A**) and FCM analysis (**B**) detecting CD269 and CD38 of Patient 10 before and after CAR-T infusion

A CIS was defined as the appearance of a new serum monoclonal that differed from the original heavy or light chain detected at diagnosis. Patient 10 was diagnosis with IgA-κ subtype MM pre-CAR-T infusion as seen in Fig. 5A, and 3 months after CAR-T infusion her MIg turned undetectable. Six months after CAR-T infusion, we observed an apparent abnormal protein band (APB) of IgG-κ type, distinct from the paraprotein present at diagnosis through SPEP analysis, without detectable MRD (Fig. 5A and B). With the passage of time, FCM did not detect bone marrow recurrence of myeloma, and this APB decreased gradually disappeared 9 months after CAR-T infusion. Our data have demonstrated that a CIS is a benign phenomenon in course of CAR-T infusion.

### Severe CRS resulted in HLH

**Fig. 6:**
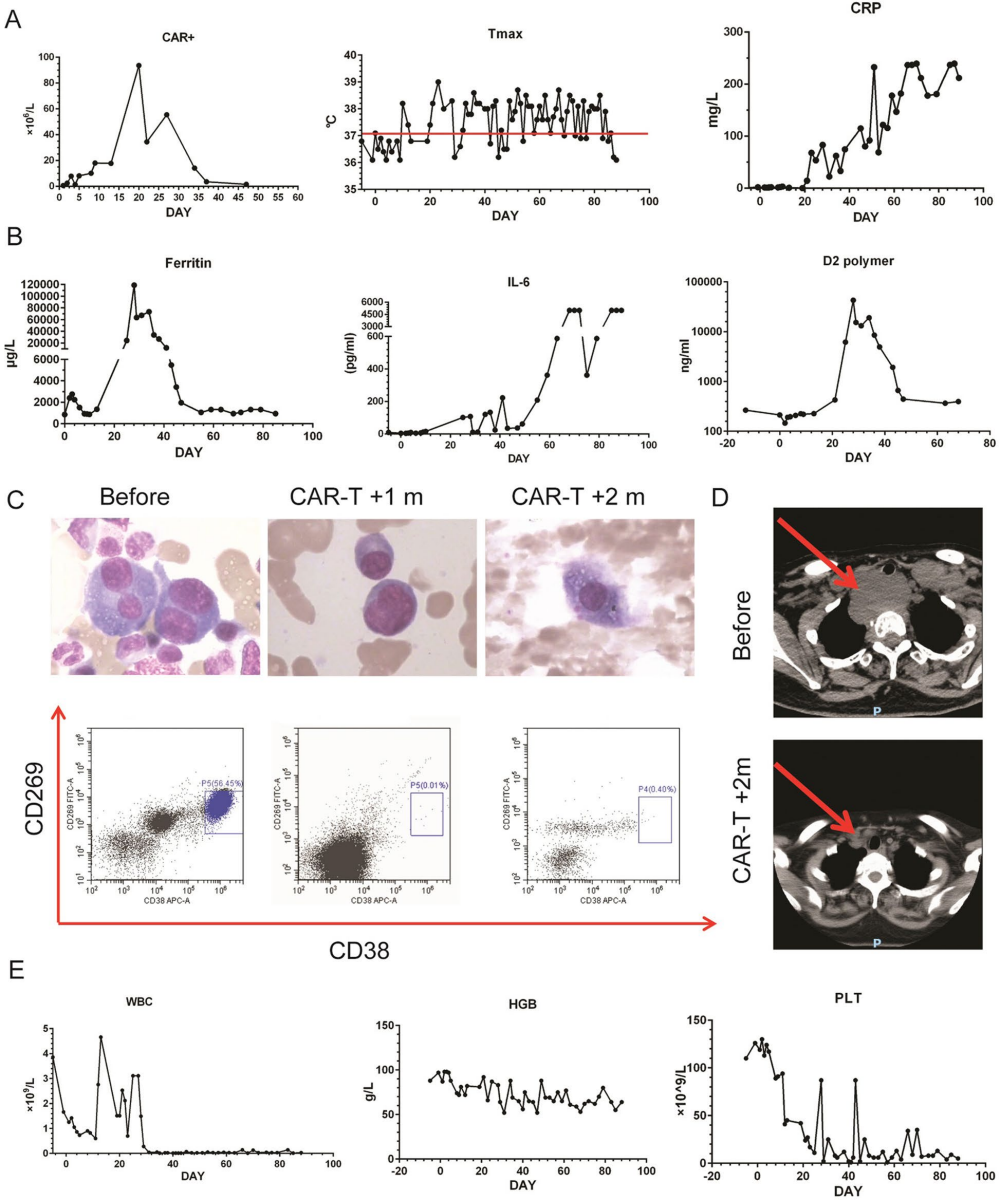
Severe CRS resulted in HLH in Patient 6. **A** CAR+ cells in PBMC of Patient 6 was tested by FCM before and after BCMA-CD38 CAR-T infusion. Curves of max value of temperature (Tmax) and CRP before and after CAR-T infusion was recorded. **B** Curves of inflammatory factors (Ferritin, IL-6, and D2 polymer) of Patient 6 before and after BCMA-CD38 CAR-T infusion. **C** Morphology and FCM analysis detecting CD269 and CD38 staining of BMC of Patient 6 before, 1 and 2 months after BCMA-CD38 CAR-T infusion. **D** The extramedullary lesion images of Patient 6 before and 2 months after BCMA-CD38 CAR-T infusion. **E** White blood cells (WBC), hemoglobin (HGB) and platelet (PLT) levels of Patient 6 before and after BCMA-CD38 CAR-T infusion

Patient 6 received 8 courses of therapy, but her disease progressed prior to enrollment. At the time of enrollment, she had IgG-λ subtype MM for 14 months. Poor prognostic cytogenetics (1q21) were detected through fluorescence in situ hybridization (FISH) (Table 1). There were 32.21% abnormal plasma cells (morphology assessment) in bone marrow (Fig. 6C), and β2-MG reached 9.66 mg/L. In addition, there was an extramedullary mass of about 6.5 × 3.4 × 6.3 cm behind the trachea (Fig. 6D). She received dose of 2.7 × 10^6^ CAR+ T cells/kg. On the day after the CAR-T cells infusion, the patient subsequently developed fatigue, tachycardia and dyspnea. By 10 days after CAR-T cells infusion, the patient had symptoms of recurrent fever, nausea, vomiting, severe ostealgia and myalgias. The patient was febrile for more than 2 months (Fig. 6A). The CRP reached a maximum of 239.5 mg/L, IL-6 reached 5000.0 pg/ml, ferritin reached 119,000.0 ng/ml, and D2 polymer reached 42,724 ng/ml (Fig. 6A, B). FCM detection also suggested a violent amplification of CAR-T cells in peripheral blood (Fig. 6A). To alleviate the severe storm of inflammatory factors, we used dexamethasone up to a total dose of 83 mg, plasma exchange and other supportive therapy.

Although the intra- and extramedullary involvement became undetectable 1 month and 2 months after CAR-T infusion, respectively (Fig. 6C and D), patient 6 experienced severe pancytopenia along with the storm of inflammatory factors. The patient remained granulocytopenia for 2 months, and the total leukocyte count was as low as 0.01 × 10^9^/L (Fig. 6E). During this neutropenic period, the patient suffered multiple carbapenem resistant enterobacteriaceae septicemia (*Stenotrophomonas maltophilia*, *Escherichia coli* and *Klebsiella pneumoniae*) and fungal infection (*Candida albicans* and *Candida tropicalis*), also resulting in recurrent fever and elevated CRP (Fig. 6A and B). Two months after CAR-T cells infusion, phagocytic cells could be observed in the bone marrow tissue, as well as absence of normal cells (Fig. 6C). The patient eventually died of septic shock. Taken together, severe CRS might lead to life threatening HLH.

## Discussion

Idel-cel and Cilta-cel had a response of 73.4 and 94.8%, respectively [11] [12]. A meta-analysis was performed to determine the safety and clinical efficacy of BCMA CAR-T cell therapy in multiple myeloma, including 640 patients in twenty-three different CAR-T-cell products. The pooled overall response rate was 80.5% (73.5–85.9%), median progression-free survival (PFS) was 12.2 (11.4–17.4) months, and CRS was observed in 80.3% (69.0–88.2%) [25]. The BCMA CAR-T has shown remarkable curative potential against advanced R/R MM, but multiple trials have also reported recurrence or treatment failures with many promising avenues for improvement [26–28]. The combination with targeting CD38 is potential to make up the shortfall. Here, we constructed a dual-targeted CAR-T capable of targeting BCMA and CD38, which showed stronger lyses effect than BCMA CAR-T cells in vitro experiments. We further confirmed a high clinical efficacy of the bispecific CAR-T with an ORR of 87.5%, 1-year PFS rates of 68.8%, and a moderate and manageable CRS of 75.0%. Despite of higher ORR, it could not be concluded that BCMA-CD38 CAR-T cells had better efficacy, due to limited cases and no randomized controlled trial study of head to head.

CD38 is low expressed on the surface of precursor lymphocytes, myeloid cells and some non-hematopoietic tissues, but highly expressed on the surface of MM cells, which motivates the development of anti-CD38 CAR-T therapy [8, 29, 30]. The widely expressed characteristic of CD38 not only makes anti-CD38 CAR-T with high affinity have strong anti-MM effects, but also target-off effects against normal tissues, indicating that CD38 is a less ideal target. We achieved a 1000-fold reduced affinity antibody, through screening from plenty of CD38 antibodies with a broad range of different affinities. Here, we designed a CAR-T carrying anti-BCMA and the low affinity anti-CD38. In addition, it is more difficult for anti-CD38 activating immunological synapse response to the target cells, due to that anti-CD38 was at external part of the anti-BCMA on the protein spatial structure. As a result, the bispecific CAR-T have the characteristics of rapid combination and reconciliation, which will make the CAR-T have no or very weak killing effect on CD38+ cells. Anti-CD38 was used for auxiliary targeting and enhancing binding to anti-BCMA, rather than killing tumor cells directly. As to the adverse event in our study, we found mild gastrointestinal symptoms, tachycardia and myalgia, which might be related with the off-target effect. However, but it is difficult to tell whether it is caused by tumor target-off effect or by CRS.

Oligoclonal immunoglobulin bands differing from those originally identified at diagnosis termed CIS has been reported in 26.4–43% patients with MM after high-dose chemotherapy or ASCT [31–34], showing that patients observed with an apparent APB had a significantly better survival. Besides, relapsed patients had disease characterized by the same type of paraprotein as that detected at diagnosis [31]. In our results, the patient 10 suffered a temporary CIS, but did not relapse. Thus, our findings indicated that CAR-T might result in APB. However, the origin of the new monoclonal bands remains largely unknown. Gene sequencing of heavy chain variable region in 7 patients with post-ASCT CIS did not show a clonal relationship to the original malignant clone isotype, highlighting nonmalignant B cells as the likely origin of CIS [35]. Multiple studies have demonstrated that greater rates of MGUS correlated with immune system hyperactivation in patients with autoimmune disease, infection, or inflammatory or allergic disorders and after myeloablative regimens in recipients of an allograft when humoral reconstitution has occurred in a clonally deregulated pattern [36, 37]. The emerging of MIg especially a new serum monoclonal is not always a sign of recurrence in MM, which should be distinguished from CIS at the same time. To the best of our knowledge, CIS after CAR-T infusion has not been reported before. Further investigations to define whether CIS might serve as an immune biomarker in CAR-T treatment and better understand the underlying mechanism will help to define a subset of patients most likely to benefit from immunotherapy.

Most CRS cases were mild and manageable, however one of them died of HLH. HLH is potentially fatal syndrome of immune hyperactivation, observed in about 1% of all patients treated with CAR-T therapy [38]. The hemocytopenia of acquired HLH is caused by excessive activation of macrophages and T lymphocytes by CRS in CAR-T treatment. Thus, CRS and HLH might belong to a similar spectrum of hyperinflammatory. Patients with HLH may have clinical features including high fever, multi-organ dysfunction, high ferritin levels of > 10,000 ng/mL, CRS grade ≥ 3, hemophagocytosis in the bone marrow or hemocytopenia [39]. Cytokine-directed therapy, corticosteroids and other supportive therapy were the preferred treatment for refractory HLH [40]. There is still no evidence to support the use of etoposide and cytarabine in CAR-T related HLH. The treatment to CAR-T cell related HLH needed more study to meet the challenge.

## Conclusions

In summary, we described here a promising strategy for the treatment of MM by targeting BCMA and CD38, which present a valid solution to the challenge of antigen escape in BCMA CAR-T therapy. Since the study was an exploratory one, later-registration clinical studies are being planned and designed, which will further demonstrate its efficacy and safety.

## Supplementary Information

**Additional file 1.** Methods 1.1 Detection of CAR-T cells by FCM. 1.2 Inclusion criteria. 1.3 Exclusion criteria.

**Additional file 2: Supplementary Fig. 1.** Consort diagram. **Supplementary Fig. 2.** The extramedullary lesion of patient 14 (A), 11 (B) and 13 (C) before and 2 months after BCMA-CD38 CAR-T infusion.

**Additional file 3: Supplementary Table 1.** Non-hematological adverse events after BCMA-CD38 CAR-T infusion. **Supplementary Table 2.** Hematological adverse events after BCMA-CD38 CAR-T infusion.

## Data Availability

The data that support this study are available from the corresponding author upon reasonable request.

## Abbreviations

APB: Abnormal protein band
ASCT: Autologous hematopoietic stem cell transplantation
BCMA: B-cell maturation antigen
BMC: Bone marrow cells
BCMA-CD38: BCMA and CD38
BOR: Best objective response
CAR-T: Chimeric antigen receptor T
CIS: Clonal Isotype Switch
CR: Complete response
CRP: C-reactive protein
CRS: Cytokine release syndrome
CT: Computed tomographic
FCM: Flow cytometry
FISH: Fluorescence in situ hybridization
HLH: Hemophagocytic lymphohistiocytosis syndrome
IL-6: Interleukin-6
IMWG: International Myeloma Working Group
ISS: International stage system
NSAIDs: Nonsteroidal anti-inflammatory drugs
MDSC: Myeloid-derived suppressor cells
MM: Multiple myeloma
MIg: Monoclonal immunoglobulin
MR: Minimal response
MRD: Minimal residual disease
MRI: Magnetic resonance imaging
ORR: Overall response rate
OS: overall survival
PBMC: Peripheral blood mononuclear cells
PD: Progression disease
PFS: Progression-free survival
PR: Partial response
R/R MM: Relapsed or refractory multiple myeloma
sCR: Stringent complete response
SD: Stable disease
SPEP: Serum protein electrophoresis
UPEP: Urine protein electrophoresis
VGPR: Very good partial response rate
β2-MG: β2 microglobulin
