# Supplementary material for: High efficacy and safety of CD38 and BCMA bispecific CAR-T in relapsed or refractory multiple myeloma
Source: J Exp Clin Cancer Res. 2022 Jan 3;41:2. doi: 10.1186/s13046-021-02214-z (PMC8722124; doi:10.1186/s13046-021-02214-z)
Supplement: Supplementary file 1 — Additional file 1. Methods 1.1 Detection of CAR-T cells by FCM. 1.2 Inclusion criteria. 1.3 Exclusion criteria. [file 13046_2021_2214_MOESM1_ESM.docx]

## Detection of CAR-T cells by FCM

The peripheral blood or bone marrow was diluted by precooled ACK lysis lysate at 1:10 and cracked at room temperature for 10 min. After the liquid was clarified and centrifuged at 1500 rpm for 10 min, the supernatant was removed. The cells washed once with PBS buffer containing 2%BSA. 2×10^6^ cells were re-suspended in 100 ul PBS, stained with 1 μg biotinylated BCMA protein (ACRO Biosystems), and incubated at 4 ℃ for 60 minutes. After washed twice with PBS buffer containing 2%BSA, the cells were added 1 μl PE labeled streptavidin (Biolegend) and incubated at 4 ℃ for 30 minutes. Then the sample was stained with 10 μl CD3-FITC (BD) and human CD45 antibody (BD) incubated in the dark at room temperature for 15 min, followed by detection by Beckman CytoFLEX flow cytometry.

## Inclusion criteria

1. Patients aged between 18 and 80 with relapsed or refractory multiple myeloma.

2. Bone marrow sample is confirmed as BCMA-positive or CD38-positive by flow cytometry or pathological examination.

3. Patients with relapsed or refractory multiple myeloma who meet the following conditions:

1) Treatment failure or disease progressed after 2 courses of standard treatment regimen;

2) Disease relapsed after chemotherapy or HSCT. Curative efficacy is little or disease progressed after 2 courses of original treatment regimen;

3) More than 60 days between last treatment and disease progression;

4) Autologous or allogeneic SCT is not available at present, or patient refuses to receive SCT;

5) Disease progression is defined as Chinese Guidelines for Diagnosis and Treatment of Multiple Myeloma (Revision in 2015). At least one of the following conditions should be met:

Serum M-protein increases >=25% (absolute increase should be >=5 g/L). If serum M protein is >=50 g/L at baseline, increase of serum M protein can be >=10 g/L; Urine M-protein increases >=25% (absolute increase should be >=200 mg/24 h); If the serum and urine M-protein are not detectable, a >=25% increase in the difference between involved and uninvolved FLC levels is required (absolute increase should be >=100 mg/L); Bone marrow plasma cell percentage increases >=25% (absolute increase should be >=10%); Size of existing bone lesions or soft tissue plasmacytomas increases by >=25%, or development of new lytic bone lesions or soft tissue plasmacytomas; Development of hypercalcemia that can be attributed to plasma cell proliferative disorder (corrected calcium is > 2.8 mmol/L or 11.5 mg/dL); Disease progression must be confirmed by 2 sequential assessments;

4. Expected survival > 12 weeks;

5. Disease is measurable, and at least one of the following conditions should be satisfied:

1) Serum M-protein is >=10 g/L;

2) 24-hour urine M-protein is >=200 mg; 3) Serum FLC is >=5 mg/dL;

4) Plasmacytomas that can be measured or evaluated by imaging

5) Bone marrow plasma cell percentage is >=20%.

6. ECOG scores 0 - 1;

7. Adequate venous access for apheresis and venous blood sampling, and no other contraindications for leukapheresis;

The above lab results should not include those obtained from continuous supportive treatment that is ongoing.

## Exclusion criteria：

1. Performed autologous or allogeneic SCT within 3 months before enrollment.

2. Asymptomatic Myeloma (Smouldering Multiple Myeloma).

3. Previous BCMA or CD38 targeted cell therapy.

4. Previous CAR-T or other lentivirus-mediated transgenic therapy.

5. Presence of uncontrollable or anti-infective fungal, bacterial, viral or other infections.

6. Patients required systemic corticosteroid therapy 5mg/d prednisone or equivalent dose of other corticosteroids) or other immunosuppressive drugs (except for adverse events) during the study period.

7. Any indwelling catheter or drainage tube (such as percutaneous nephrostomy, indwelling catheter, bile drainage tube or pleural/peritoneal/pericardial catheter) is present. The use of a dedicated central venous catheter is allowed.

8. A history or disease of the central nervous system, such as seizure disease, cerebral ischemia/hemorrhage, dementia, cerebellar disease, or any autoimmune disease involving the central nervous system.

9. Presence of clinically significant cardiovascular disease, such as uncontrolled or symptomatic arrhythmias, congestive heart failure, or any heart function grade 3 (moderate) or grade 4 (severe) heart disease (according to New York Heart Association function classification method). A history of myocardial infarction, angioplasty or stenting, unstable angina or other clinically significant heart disease in the 12 months prior to enrollment.

10. Patients with primary immunodeficiency diseases (such as severe combined immunodeficiency diseases, etc.).

11. Patients with a history of pulmonary embolism.

12. A history of severe hypersensitivity of the main therapeutic drugs in this study (including fludarabine, cyclophosphamide and mesoderma used during pretreatment, as well as anti-IL-6 monoclonal antibody, ruxotinib and anti-infection drugs used in the prevention and treatment of CRS).

13. Pregnant or lactating women.

14. Male and female subjects who are not willing to take birth control measures within 6 months after signing the consent form and completion of car-t administration.

15. Patients are participating in other intervention studies.

16. Patients are unlikely to complete all protocol required study visits or procedures, including follow-up visits or compliance with study participation requirements in the investigator's judgment.

17. A history of autoimmune diseases (such as Crohn's disease, rheumatoid arthritis, systemic lupus erythematosus) in the past 2 years that resulted in end-organ damage or required systemic immunosuppressive/disease-regulating drugs.
