# Supplementary material for: High efficacy and safety of CD38 and BCMA bispecific CAR-T in relapsed or refractory multiple myeloma
Source: J Exp Clin Cancer Res. 2022 Jan 3;41:2. doi: 10.1186/s13046-021-02214-z (PMC8722124; doi:10.1186/s13046-021-02214-z)
Supplement: Supplementary file 2 — Additional file 2: Supplementary Fig. 1. Consort diagram. Supplementary Fig. 2. The extramedullary lesion of patient 14 (A), 11 (B) and 13 (C) before and 2 months after BCMA-CD38 CAR-T infusion. [file 13046_2021_2214_MOESM2_ESM.docx]

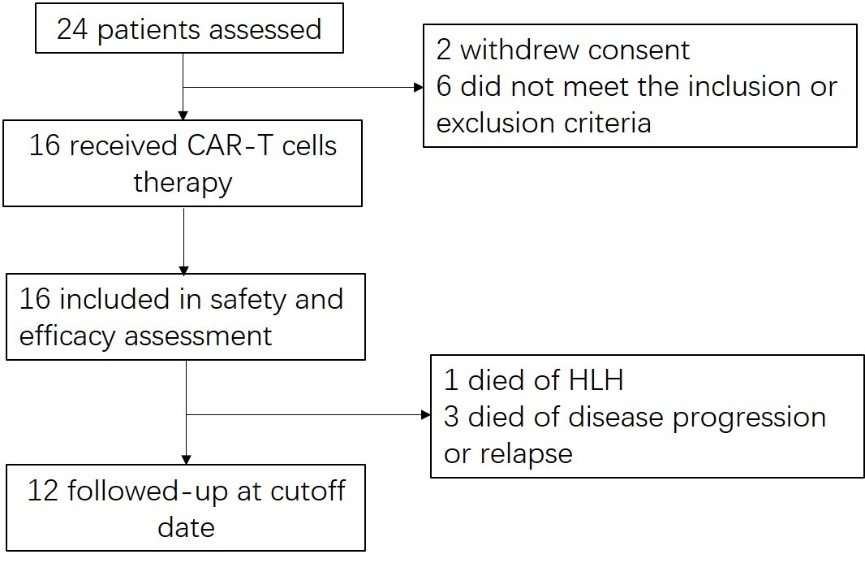


**Supplementary Fig. 1**. Consort diagram.


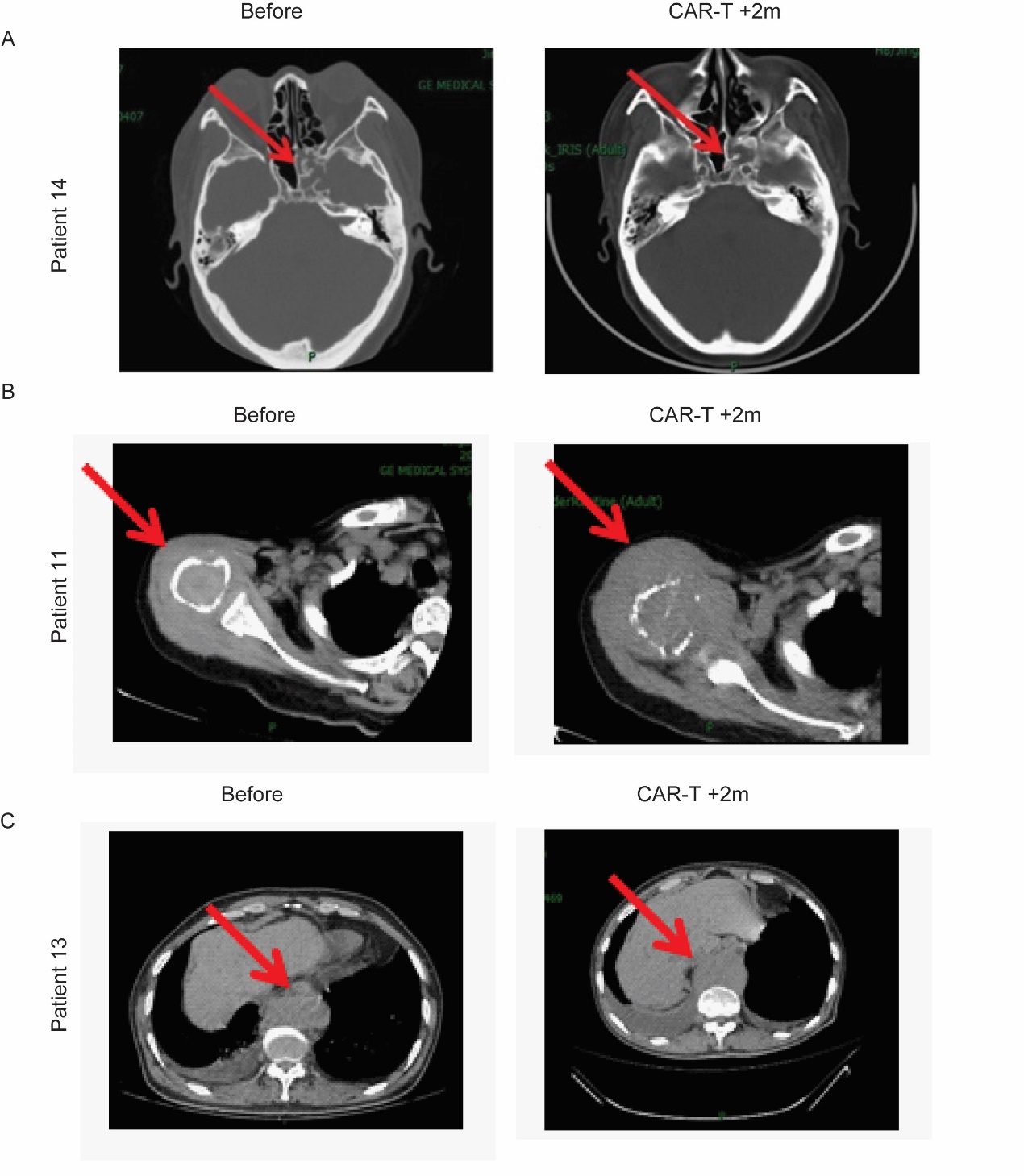


**Supplementary Fig. 2**. The extramedullary lesion of patient 14 **(A)**, 11 **(B)** and 13 **(C)** before and 2 months after BCMA-CD38 CAR-T infusion.
