# Supplementary material for: High efficacy and safety of CD38 and BCMA bispecific CAR-T in relapsed or refractory multiple myeloma
Source: J Exp Clin Cancer Res. 2022 Jan 3;41:2. doi: 10.1186/s13046-021-02214-z (PMC8722124; doi:10.1186/s13046-021-02214-z)
Supplement: Supplementary file 3 — Additional file 3: Supplementary Table 1. Non-hematological adverse events after BCMA-CD38 CAR-T infusion. Supplementary Table 2. Hematological adverse events after BCMA-CD38 CAR-T infusion. [file 13046_2021_2214_MOESM3_ESM.docx]

**Supplementary Table 1.** Non-hematological adverse events after BCMA-CD38 CAR-T infusion.

| NO. | Adverse Events | start time | persistent time (d) | CRS grade | Treatment |
| --- | --- | --- | --- | --- | --- |
| 1 | fever | d3 | 2 | 2 | NSAIDS, anti-infection |
|  | myalgias |  |  |  |  |
|  | fatigue |  |  |  |  |
|  | dissociative arthralgia |  |  |  |  |
|  | upper respiratory tract infection |  |  |  |  |
| 2 | ostealgia | d1 | 25 | 3 | NSAIDS, steroids, anti-infection, blood product infusion and other supportive therapy |
|  | fatigue |  |  |  |  |
|  | fever |  |  |  |  |
|  | coagulopathy |  |  |  |  |
|  | dissociative arthralgia |  |  |  |  |
| 3 | fatigue | d3 | 3 | 3 | NSAIDS, anti-infection, and oxygen therapy |
|  | fever |  |  |  |  |
|  | dissociative arthralgia |  |  |  |  |
|  | myalgias |  |  |  |  |
|  | lung infection |  |  |  |  |
|  | Headache |  |  |  |  |
| 4 | No |  |  | 0 | N/A |
| 5 | myalgias | d5 | 2 | 1 | No |
|  | dissociative arthralgia |  |  |  |  |
|  | digestive infection |  |  |  |  |
| 6 | fever | d0 | 45 | 5 | NSAIDS, steroids, anti-infection, blood product infusion, plasma exchange and other supportive therapy. |
|  | dyspnea |  |  |  |  |
|  | ostealgia |  |  |  |  |
|  | fatigue |  |  |  |  |
|  | myalgias |  |  |  |  |
|  | gastrointestinal symptoms |  |  |  |  |
|  | HLH |  |  |  |  |
|  | septicemia |  |  |  |  |
|  | focal pain |  |  |  |  |
| 7 | No |  |  | 0 | N/A |
| 8 | tachycardia | d15 | 12 | 2 | NSAIDS, anti-infection |
|  | fever |  |  |  |  |
|  | fatigue |  |  |  |  |
|  | numb |  |  |  |  |
|  | coagulopathy |  |  |  |  |
|  | ostealgia |  |  |  |  |
| 9 | dizzy | d12 | 4 | 2 | NSAIDS, anti-infection |
|  | myalgias |  |  |  |  |
|  | fever |  |  |  |  |
| 10 | ostealgia | d1 | 13 | 3 | NSAIDS, anti-infection, liver function impairment, and other supportive therapy |
|  | myalgias |  |  |  |  |
|  | fatigue |  |  |  |  |
|  | liver dysfunction |  |  |  |  |
|  | lung infection |  |  |  |  |
|  | fever |  |  |  |  |
| 11 | No |  |  | 0 | N/A |
| 12 | fever | d3 | 11 | 3 | NSAIDS, steroids, anti-infection, and other supportive therapy |
|  | fatigue |  |  |  |  |
|  | dizzy |  |  |  |  |
|  | lung infection |  |  |  |  |
| 13 | No |  |  | 0 | N/A |
| 14 | gastrointestinal symptoms | d1 | 13 | 1 | Proton-pump inhibitor (PPI), NSAIDS |
|  | myalgias |  |  |  |  |
|  | gastrointestinal symptoms |  |  |  |  |
|  | dissociative arthralgia |  |  |  |  |
|  | focal pain |  |  |  |  |
|  | numb |  |  |  |  |
| 15 | focal pain | d1 | 10 | 1 | NSAIDS, painkiller |
|  | dyspnea |  |  |  |  |
|  | fever |  |  |  |  |
|  | fatigue |  |  |  |  |
| 16 | fever | d7 | 3 | 2 | NSAIDS, painkiller, anti-infection |
|  | myalgias |  |  |  |  |

cytokine release syndrome, CRS; Not available, N/A

**Supplementary Table 2.** Hematological adverse events after BCMA-CD38 CAR-T infusion.

|  | Grade 1-2 | Grade 3 | Grade 4 | Grade 5 |
| --- | --- | --- | --- | --- |
| Leukopenia | 1 (6.3%) | 10 (62.5%) | 4 (25.0%) | 1 (6.3%) |
| Anemia | 7 (43.8%) | 5 (31.3%) | 1 (6.3%) | 0 |
| Thrombocytopenia | 2 (12.5%) | 2 (12.5%) | 2 (12.5%) | 0 |
